# Video polysomnographic findings in non‐rapid eye movement parasomnia

**DOI:** 10.1111/jsr.12772

**Published:** 2018-10-08

**Authors:** Panagis Drakatos, Lucy Marples, Rexford Muza, Sean Higgins, Alexander Nesbitt, Eptehal M. Dongol, Raluca Macavei, Valentina Gnoni, Laura Perez Carbonell, Iain Duncan, Adam Birdseye, Sakina Dastagir, Ivana Rosenzweig, David O’Regan, Adrian J. Williams, Guy D. Leschziner, Brian D. Kent

**Affiliations:** ^1^ Sleep Disorders Centre Guy’s Hospital London UK; ^2^ Sleep and Brain Plasticity Centre, Department of Neuroimaging IOPPN, King’s College London London UK; ^3^ King’s College London London UK; ^4^ Department of Clinical Neurosciences King’s College London London UK

**Keywords:** NREM parasomnia, OSA, PLMS, polysomnography

## Abstract

Although video polysomnography (vPSG) is not routinely recommended for the evaluation of typical cases of non‐rapid eye movement (NREM) parasomnias, it can aid diagnosis of unusual cases, other sleep disorders and complicated cases with REM behaviour disorder (RBD), and in differentiating parasomnias from epilepsy. In this study, we aimed to assess vPSG findings in consecutive patients with a clinical diagnosis of NREM‐parasomnia covering the whole phenotypic spectrum. Five hundred and twelve patients with a final diagnosis of NREM parasomnia who had undergone vPSG were retrospectively identified. vPSGs were analysed for features of NREM parasomnia and for the presence of other sleep disorders. Two hundred and six (40.0%) patients were clinically diagnosed with sleepwalking, 72 (14.1%) with sleep terrors, 39 (7.6%) with confusional arousals, 15 (2.9%) with sexsomnia, seven (1.4%) with sleep‐related eating disorder, 122 (23.8%) with mixed phenotype, and 51 (10.0%) with parasomnia overlap disorder (POD). The vPSG supported the diagnosis of NREM parasomnia in 64.4% of the patients and of POD in 98%. In 28.9% of the patients, obstructive sleep apnea (OSA) or/and periodic limb movements during sleep (PLMS) were identified, most commonly in older, male, sleepy and obese patients. vPSG has a high diagnostic yield in patients with NREM parasomnia and should be routinely performed when there is diagnostic doubt, or in patients where there is a suspicion of OSA and PLMS.

## 1 INTRODUCTION

The non‐rapid eye movement (NREM) parasomnias are a family of disorders characterized by unwanted behaviours or experiences, which primarily arise from slow wave sleep (NREM3). They include sleepwalking, sleep terrors, confusional arousals, sexsomnia and sleep‐related eating disorder (SRED) (American Academy of Sleep Medicine 2013; Auger, 2006; Muza, Lawrence, & Drakatos, 2016; Schenck & Mahowald, 1994). The diagnosis of NREM parasomnia is often made on the basis of clinical history, although video‐polysomnographic (vPSG) recordings may be required in assessing complicated or difficult cases, particularly when a diagnosis of epilepsy is suspected. (Bisulli et al., 2011; Derry et al., 2006; Fois, Wright, Sechi, Walker, & Eriksson, 2015; Zucconi et al., 1997). They may also reveal features suggestive of NREM parasomnia, such as abrupt arousals from NREM3 sleep, or may confirm the diagnosis by capturing relevant behaviours arising from NREM3 (Aldrich & Jahnke, 1991; Blatt, Peled, Gadoth, & Lavie, 1991; Fois et al., 2015; Kavey, Whyte, Resor, Jr, & Gidro‐Frank, 1990).

The current American Academy of Sleep Medicine (AASM) guidelines on the use of vPSG in parasomnia suggest that it be reserved for patients with violent or potentially injurious behaviours, cases where there is a failure to respond to therapy, or cases with an atypical or forensic presentation (Kapur et al., 2017). These guidelines do not comment on the identification of concomitant sleep pathology in patients with parasomnia, such as obstructive sleep apnea (OSA) and periodic limb movements during sleep (PLMS). Identifying these may be meaningful as they may serve as precipitating factors for NREM parasomnia behaviours, facilitating different approaches to the treatment of parasomnia patients (Fois et al., 2015; Guilleminault et al., 2005).

Our current understanding of sleep and sleep pathology in NREM parasomnia is mainly based on relatively small case series (Brion et al., 2012; Espa, Dauvilliers, Ondze, Billiard, & Besset, 2002; Guilleminault et al., 2005; Guilleminault, Moscovitch, Yuen, & Poyares, 2002), observational studies with polysomnographic recordings (Fois et al., 2015; Guilleminault, Palombini, Pelayo, & Chervin, 2003), and epidemiological studies that did not involve polysomnographic assessment of the patients (Ohayon, Mahowald, Dauvilliers, Krystal, & Leger, 2012). Many of the studies published to date have focused on individual NREM parasomnia phenotypes. Similarly, relatively little is known about vPSG findings in patients with parasomnia overlap disorder (POD), wherein NREM parasomnia and REM behaviour disorder (RBD) coexist, something reported in up to 28% of patients with sleepwalking or sleep terrors (Schenck & Howell, 2013; Schenck, Boyd, & Mahowald, 1997).

The aim of this study was to assess the findings of vPSG performed in a large cohort of patients with a diagnosis of NREM parasomnia with or without coexisting RBD. We aimed to evaluate the diagnostic utility of vPSG both in NREM parasomnia and POD, particularly in the identification of other sleep disorders that may serve as precipitants of parasomnia behaviours.

## 2 MATERIALS AND METHODS

### 2.1 Patient selection

Using an internal sleep laboratory database, we retrospectively identified adult patients with a final diagnosis of NREM parasomnia, following a consultation with an experienced sleep physician at the Sleep Disorders Centre, Guy's and St Thomas' Hospitals, London, UK, over a period of 7.5 years (between June 2008 and December 2015).

**Figure 1:**
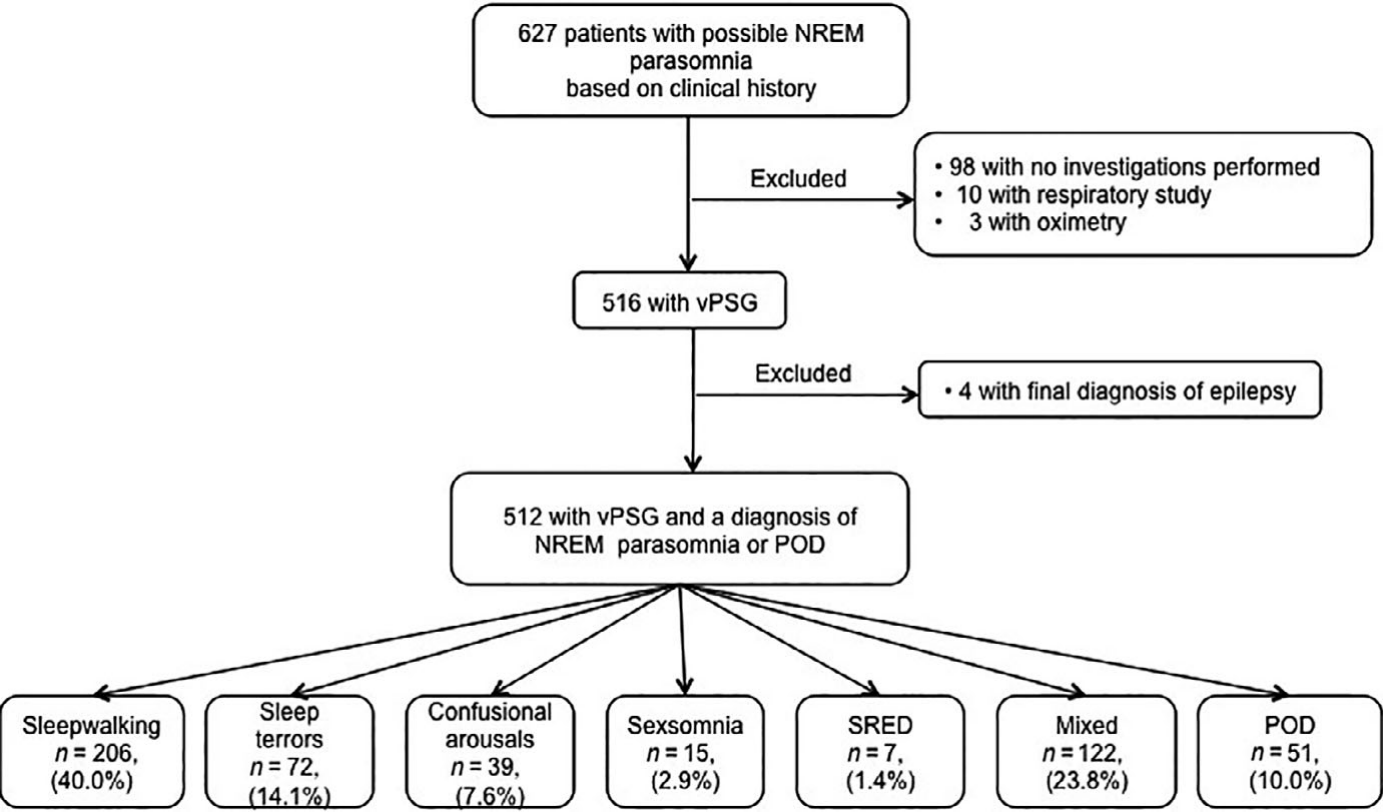
Flow diagram of the studied cohort. Percentages express the prevalence of each diagnostic group within the cohort of 512 patients. vPSG, video polysomnography; SRED, sleep‐related eating disorder; Mixed, mixed phenotypes of NREM parasomnia; NREM, non‐rapid eye movement sleep; POD, parasomnia overlap disorder

All cases were retrospectively reviewed, and patients with a final diagnosis of NREM parasomnia or POD, based on International Classification of Sleep Disorders 3rd edition (ICSD‐3) criteria (AASM 2013), who had undergone vPSG assessment, were included in the analysis and were divided into six major categories (Figure 1). Patients with coexisting phenotypes of NREM parasomnia were grouped as mixed parasomnia. None of the patients included had received a prior diagnosis or treatment for NREM parasomnia, POD or other sleep disorders. Demographics, Epworth sleepiness scale (ESS) scores and clinical factors, including medication use, were obtained from patients’ medical notes. A standardized diagnostic approach was shared between clinicians with the intention to order vPSG for all patients with clinical presentation of NREM parasomnia.

### 2.2 Sleep study methodology

Inpatient vPSG was performed using a standard 10–20 EEG montage for all patients, with sleep stages scored according to the standard criteria of the AASM. Extended montage EEG with additional leads (17 versus 6 for standard montage) was applied to patients with a clinical suspicion of epilepsy. Continuous recordings included electrooculography, electrocardiography, submental and leg electromyography, pulse oximetry, nasal pressure cannulae and respiratory inductance plethysmography, with chest and abdominal belts (Berry et al., 2015 2015). Time‐synchronized video recordings were also performed, while a multidirectional microphone placed above the bed captured audio recordings. In patients requiring multiple sleep latency testing for diagnostic purposes of concomitant sleep disorders, standard guidelines were applied (Carskadon et al., 1986).

The scoring of arousals and of REM without atonia (RWA) was based on the AASM guidelines, and the arousal index (AI) is reported as the number of arousals per hour of sleep. The diagnosis of RBD required evidence of dream enactment during REM sleep on vPSG or a characteristic clinical history alongside the presence of RWA (sustained muscle activity and/or excessive transient muscle activity in the chin or limb EMG during REM sleep). Apneas, hypopneas and periodic limb movements in sleep (PLMS) were scored following standard AASM definitions to determine the apnea–hypopnea index (AHI) and PLM index (PLMI), using as cut‐offs 5 events/hr and 15 events/hr for the diagnosis of obstructive sleep apnea (OSA) and PLMS, respectively (Berry et al., 2015 2015). Additional analyses were performed using the more clinically relevant AHI cut‐offs of moderate–severe OSA (i.e. AHI ≥15 events/hr) and OSA syndrome (OSAS; i.e. AHI ≥5 events/hr with daytime sleepiness). The presence of spontaneous arousals from NREM3 with typical NREM parasomnia behaviours (NREM3‐A), spontaneous arousals accompanied by more subtle behaviours such as raising the head, sympathetic activation, such as tachycardia, or rhythmic delta activity on EEG (NREM‐a), were recorded.

All cases of concomitant diagnosis of hypersomnia of central origin were retrospectively reviewed and diagnosis was made *de novo* according to ICSD‐3 criteria (AASM 2013).

### 2.3 Priming and precipitating factors

Medical records were searched for priming factors associated with NREM parasomnia, including sleep deprivation, stress, insomnia, hyperthyroidism, migraines, history of head injury, encephalitis and stroke, and drugs including lithium, phenothiazines, anticholinergic agents, hypnotic drugs and sodium oxybate. Sleep deprivation was captured through sleep dairies or actigraphy, and stress was recorded based on subjects' reports. The diagnosis of insomnia was made based on the existing ICSD criteria at the time of the diagnosis.

Potential precipitating factors, including external stimuli such as noise, light and bed partner's movement, and internal stimuli such as OSA and PLMS, were extracted from patients' notes and vPSG.

### 2.4 Statistical analysis

Data are reported as mean ± standard deviation if not otherwise indicated. In analyses of demographic, clinical and sleep parameters between parasomnia subtypes, comparisons between groups were performed using the Kruskal–Wallis test with Dunn's multiple comparison test when needed for continuous variables, whereas chi‐squared with Cramer's V product was used for comparisons between nominal variables. To estimate the likelihood of concomitant sleep‐disrupting pathology (i.e. OSA or PLMS), a logistic regression model was built incorporating parasomnia phenotypes, age, gender, obesity and subjective daytime sleepiness, with the presence or absence of sleep‐disrupting pathology as the dependent variable. A value of *p* < 0.05 was considered to be statistically significant. IBM SPSS Statistics V24.0 (SPSS, Chicago, IL, USA) was used for all statistical analysis.

## 3 RESULTS

### 3.1 Diagnosis and priming factors

Based on clinical history, 627 patients with a possible diagnosis of NREM parasomnia or POD were identified. Of those, 512 had undergone vPSG with an ultimate diagnosis of NREM parasomnia, which could overlap with RBD (Figure 1). The majority (*n* = 98; 88.3%) of the patients excluded from this report did not have any investigations, primarily (95%) because of patient preference or existing investigations from elsewhere. Sleepwalking was the most commonly identified phenotype, and the majority of patients with a mixed phenotype (23.8% of the cohort) had sleepwalking and sleep terrors. The most common NREM parasomnias within the POD group (10.0% of the cohort) were sleepwalking and confusional arousals (Table 1); 21.5% (17/79) of the patients who had confusional arousals irrespective of the presence of other concomitant NREM parasomnia (isolated, *n* = 39; mixed, *n* = 23; POD, *n* = 17) also had RBD.

**Table 1:** Distribution of NREM parasomnia phenotypes within the mixed phenotype and parasomnia overlap disorder groups

NREM parasomnia phenotypes	Diagnosis	
	Mixed *n* = 122	POD *n* = 51
Sleepwalking	111 (91.0%)	26 (51.0%)
Sleep terrors	86 (70.5%)	10 (19.6%)
Confusional arousals	23 (18.9%)	17 (33.3%)
Sexsomnia	15 (12.3%)	1 (2.0%)
SRED	17 (13.9%)	0 (0%)

Note

NREM: non‐rapid eye movement sleep; POD: parasomnia overlap disorder; SRED: sleep‐related eating disorder; Mixed: mixed phenotypes of NREM parasomnia.

Patients in the mixed and POD groups could present with more than one NREM phenotype.

Patients diagnosed with POD were the oldest among the groups. There was a male predominance in the sexsomnia group, whereas females predominated in the sleep terror and SRED groups. No differences in BMI and ESS scores were identified between phenotypes (Table 2).

**Table 2:** Demographics and priming factors of the studied groups

Demographics	Diagnosis							
	Sleepwalking *n* = 206	Sleep terrors *n* = 72	Confusional arousals *n* = 39	Sexsomnia *n* = 15	SRED *n* = 7	Mixed *n* = 122	POD *n* = 51	*p* value
Age (year)	38.5 ± 11.3	38.9 ± 14.3	42.9 ± 14.3	36.9 ± 8.0	43.8 ± 18.2	37.2 ± 10.6	45.1 ± 14.9	0.023^a^
Gender (male, %)	52.4	40.3	53.8	86.7	28.6	49.2	62.7	0.017^b^
BMI (kg/m^2^)	27.3 ± 5.1	25.7 ± 4.6	29.1 ± 7.2	25.9 ± 5.0	26.2 ± 5.3	27.4 ± 4.7	27.2 ± 4.0	0.203
ESS	9.6 ± 5.8	9.1 ± 4.4	11.5 ± 6.6	9.9 ± 6.5	8.5 ± 6.0	8.7 ± 5.2	10.5 ± 4.8	0.201
Priming factors								
Yes/no	70/136	32/40	18/21	3/12	5/2	40/82	16/35	0.093
Drugs	1	2	1	0	0	1	0	
Insomnia	7	3	1	2	1	6	4	
Migraines/hyperthyroidism	1	3	2	0	0	4	1	
Sleep deprivation	27	7	5	1	0	9	5	
Stress	34	20	9	1	4	20	7	

Notes

POD: parasomnia overlap disorder; SRED: sleep‐related eating disorder; Mixed: mixed phenotypes of NREM parasomnia; ESS: Epworth sleepiness scale; BMI: body mass index.

Patients may have had more than one priming factor. Independent samples Kruskal–Wallis test with Dunn's multiple comparison test was used for comparison of continuous variables between groups. Chi‐squared with Cramer's V product was used for association between nominal variables.

a Sleep terrors versus POD (*p* = 0.009), and Mixed versus POD (*p* = 0.004) using independent samples Kruskal–Wallis test with Dunn's multiple comparison test

b Chi‐squared with Cramer's V product.

In 35.9% of the patients, a priming factor was identified. The most common of these was stress (52.1%), followed by sleep deprivation (29.3%), insomnia (13.0%), migraines (4.9%), drugs (2.7%; including lithium, hypnotic drugs and sodium oxybate) and hyperthyroidism (1%). Priming factors were most frequently reported in SRED (71.4%), followed by confusional arousals (46.1%), sleep terrors (44.4%), sleepwalking (33.9%), the mixed group (32.8%), POD (31.4%) and sexsomnia (20.0%), although these differences did not reach statistical significance (Table 2).

### 3.2 Polysomnography results

No significant differences between phenotypes were observed in the vPSG sleep macrostructure (Table 3). At least one precipitating factor was identified on vPSG in 28.9% of patients (Table 3): 113 (22.0%) patients were diagnosed with OSA (AHI, 18.6 ± 15.5 events/hr) and 57 (11.1%) with PLMS (PLMI, 33.2 ± 23.6 events/hr); no significant difference was observed between parasomnia phenotypes (*p* > 0.05, Table 3); 9.2% of patients had moderate–severe OSA, whereas 11.1% had OSAS. Again, no significant difference in the prevalence of moderate–severe OSA, or of OSAS, was observed between phenotypes. In multivariate logistic regression, age >50 years, obesity, male sex and subjective daytime sleepiness were identified as independent predictors of concomitant OSA and/or PLMS (Table 4). Calculating the dependent variable of potentially sleep‐disrupting pathology using moderate–severe OSA or OSAS instead of OSA led to similar results.

**Table 3:** Polysomnographic findings and the prevalence of sleep‐disrupting pathology in patients with NREM parasomnia or parasomnia overlap disorder

Sleep parameters	Diagnosis							
	Sleepwalking *n* = 206	Sleep terrors *n* = 72	Confusional arousals *n* = 39	Sexsomnia *n* = 15	SRED *n* = 7	Mixed *n* = 122	POD *n* = 51	*p* value
TST (min)	377.9 ± 64.7	374.1 ± 69.7	367.8 ± 84.3	383.2 ± 78.7	391.3 ± 63.4	379.9 ± 72.8	369.7 ± 69.0	0.933
WASO (min)	62.9 ± 62.0	70.6 ± 54.8	76.8 ± 57.9	45.1 ± 23.4	58.3 ± 37.3	62.9 ± 44.2	66.5 ± 43.0	0.182
SO (min)	17.7 ± 14.3	25.1 ± 28.3	22.2 ± 13.2	16.9 ± 13.7	13.5 ± 4.1	22.5 ± 23.2	23.8 ± 19.3	0.064
SE (%)	82.8 ± 14.3	80.0 ± 13.2	79.2 ± 15.9	85.8 ± 7.2	82.3 ± 10.4	82.4 ± 12.2	81.1 ± 12.7	0.186
AI (events/hr)	18.8 ± 11.5	18.8 ± 8.8	16.7 ± 7.6	18.9 ± 8.5	20.3 ± 7.2	19.0 ± 12.1	17.8 ± 8.2	0.875
NREM1 % of TST	8.8 ± 7.6	7.6 ± 4.8	11.4 ± 8.7	7.6 ± 4.0	8.4 ± 3.5	8.7 ± 6.5	9.3 ± 7.3	0.295
NREM2 % of TST	44.1 ± 10.2	45.2 ± 10.5	44.2 ± 10.9	42.6 ± 9.2	52.7 ± 12.4	44.9 ± 10.9	40.4 ± 10.9	0.079
NREM3 % of TST	27.7 ± 10.1	27.7 ± 9.5	26.5 ± 10.9	28.9 ± 11.3	25.8 ± 7.1	27.8 ± 11.1	29.9 ± 10.4	0.622
REM % of TST	19.3 ± 5.8	19.4 ± 5.6	18.5 ± 6.9	19.1 ± 7.0.8	17.0 ± 5.4	18.8 ± 6.4	210.9 ± 9.4	0.669
AHI (events/hr)	5.1 ± 7.6	5.0 ± 14.2	7.2 ± 10.1	6.7 ± 8.3	6.5 ± 10.9	5.1 ± 10.9	6.7 ± 11.4	0.292
PLMI (events/hr)	6.0 ± 11.4	4.5 ± 5.5	6.2 ± 11.1	10.2 ± 14.0	12.7 ± 24.4	6.8 ± 15.8	9.3 ± 14.8	0.504
Sleep pathology								
OSA, *n* (%)	45 (21.8)	12 (16.7)	13 (33.3)	7 (46.7)	2 (28.6)	21 (17.2)	13 (25.5)	0.072
PLMS, *n* (%)	20 (9.7)	4 (5.6)	6 (15.4)	4 (26.7)	1 (14.3)	12 (9.8)	10 (19.6)	0.087
OSA and/or PLMS, *n* (%)	55 (26.7)	15 (20.8)	17 (43.5)	9 (60.0)	2 (28.5)	30 (24.6)	20 (39.2)	0.187
NT1/NT2/IH, *n*	1/1/5	0/0/1	0/2/1	0 (0)	0 (0)	0 (0)	0 (0)	0.063
vPSG markers								
NREM‐A, *n* (%)	63 (30.5)	27 (37.5)	10 (25.6)	2 (13.3)	1 (14.2)	46 (37.7)	12 (23.5)	0.182
NREM‐a, *n* (%)	59 (28.6)	20 (27.8)	12 (30.7)	9 (60.0)	2 (28.5)	47 (38.5)	20 (39.2)	0.365
RWA, *n* (%)	16 (7.7)	6 (8.3)	5 (12.8)	7 (46.6)	0 (0)	15 (12.3)	50 (98.0)	<0.001^a^

Mixed, mixed phenotypes of NREM parasomnia; SRED, sleep‐related eating disorder; POD, parasomnia overlap disorder; OSA, obstructive sleep apnea; PLMS, periodic limb movements during sleep; NT1, narcolepsy type 1; NT2, narcolepsy type 2; IH, idiopathic hypersomnia; vPSG, video‐polysomnography; NREM, non‐rapid eye movement; NREM‐A, spontaneous arousals from NREM3 with typical parasomnia behaviours; NREM‐a, spontaneous arousals accompanied by more subtle behaviours, such as raising the head, sympathetic activation, such as tachycardia, or rhythmic delta activity on EEG; RWA, REM without atonia; TST, total sleep time; WASO, wake after sleep onset time; SO, sleep onset; SE, sleep efficiency; AI, arousal index; AHI, apnea–hypopnea index; PLMI, periodic limb movement index.

Independent samples Kruskal–Wallis test with Dunn's multiple comparison test was used for comparison of continuous variables between groups. Chi‐squared with Cramer's V product was used for association between nominal variables.

a Chi‐squared with Cramer's V product test.

**Table 4:** Likelihood of concomitant sleep‐disrupting pathology

Variable	Odds ratio	95% confidence interval	*p* value
Age >50 years	6.51	3.39–12.48	<0.001
BMI >30 kg/m^2^	2.92	1.55–5.50	0.001
ESS score >10	1.92	1.10–3.43	0.022
Male	3.12	1.76–5.52	<0.001
POD	1.01	0.41–2.49	0.98
Sleepwalking	1.7	0.31–9.26	0.54
Confusional arousals	2.12	0.42–10.71	0.36
Sleep terrors	0.91	0.16–5.00	0.91
Sleep eating	1.07	0.13–8.61	0.95
Sexsomnia	4.07	0.56–29.50	0.164
Mixed parasomnia	0.93	0.13–6.32	0.93

BMI: body mass index; ESS: Epworth sleepiness scale; POD: parasomnia overlap disorder; OSA: obstructive sleep apnea; NREM: non‐rapid eye movement.

Logistic regression model with the presence of OSA and/or PLMS as the dependent variable, and age, gender, obesity, subjective daytime sleepiness and NREM parasomnia phenotypes as independent variables.

vPSG confirmed or supported the diagnosis of NREM parasomnia in 64.4% (330/512) of the patients and of POD in 9.8% (50/512); 33.0% (169/512) of the patients exhibited NREM‐a supportive of an NREM parasomnia diagnosis, and 31.4% (161/512) exhibited abnormal behaviours arising from slow wave sleep (NREM‐A), with no difference seen between NREM parasomnia phenotypes (Table 3). The vast majority of the overt parasomnia behaviours (156/161, 96.9%) in this cohort were confusional arousals, with the remainder manifesting as sleep terrors. Neither OSA nor PLMS predicted the presence of NREM‐A or NREM‐a. Of the 148 (28.9%) patients with OSA and/or PLMS, 86 (58%) had either overt parasomnia behaviours or sudden arousals from N3 sleep with more subtle behaviours. Only nine (10.5%) of these patients had OSA/PLMS episodes reported as immediately preceding an arousal. No differences were seen between subtypes of NREM parasomnia (*p* = 0.271). Fifty‐one (9.9%) of the patients were receiving medications that could affect the diagnostic yield of the vPSG, including 31/512 (6%) on serotonin reuptake inhibitors, two (0.3%) on serotonin–norepinephrine reuptake inhibitors, 13 (2.5%) on tricyclic antidepressants, three (0.5%) on benzodiazepines and two (0.3%) on zaleplon (Fois et al., 2015). No correlation was found between the administration of the drugs above and the occurrence of NREM‐a, NREM‐A, or RWA and RBD, on the vPSG (*p* > 0.05).

Unsurprisingly, 98.0% (50/51) of the patients with POD exhibited RWA. In one case, the diagnosis of RBD was based on a typical RBD manifestation captured during vPSG despite lack of RWA. The incidental finding of RWA, that is, without polysomnographic manifestations or clinical history of RBD, was present in 10.7% (49/457) of the rest of the patients without POD and was most common in the sexsomnia group (46.6%, *p* < 0.001) (Table 3).

## 4 DISCUSSION

To our knowledge, this is the largest report to date of vPSG findings in patients with a final clinical diagnosis of an NREM parasomnia. It is also one of the few reports evaluating the full spectrum of NREM parasomnias, including overlap parasomnia. The principal findings in this cohort were that there is a significant likelihood of concomitant sleep‐disrupting pathology in parasomnia patients, that vPSG can be a useful adjunct in confirming the diagnosis of NREM parasomnia, and that priming factors that increase the likelihood of parasomnia events occurring are identifiable in a sizeable minority of patients.

Sleep‐disrupting pathology, such as OSA and PLMS, has been reported in NREM parasomnia patients and is considered a potential precipitant for parasomnia behaviours, causing arousals and sleep fragmentation (Espa et al., 2002; Fois et al., 2015; Guilleminault et al., 2005, 2003 ; Kothare & Kaleyias, 2010; Ohayon et al., 2012). Prior sleep laboratory‐based studies have suggested that sleep studies may identify OSA and/or PLMS in a high proportion of patients. For example, in a cohort of 124 patients with NREM parasomnia, 21% had sleep‐disrupting pathology seen on vPSG (Fois et al., 2015), and a study of 50 sleepwalkers undergoing polysomnography found an AHI >5 events/hr in 24% (Guilleminault et al., 2005). At a population level, a diagnosis of OSA appears to confirm a nearly four‐fold increase in the likelihood of frequent sleepwalking (Ohayon et al., 2012). Our finding within a very large parasomnia cohort (that 28.9% of our patients had OSA and/or PLMS) is strikingly consistent with prior reports, and suggests that formal sleep studies should be considered, particularly in older, male, obese or sleepy patients presenting with an apparent NREM parasomnia. This is particularly the case in patients with sexsomnia, confusional arousals and POD, amongst whom there was a prevalence of physical pathology of almost 40% or above (Fois et al., 2015), and in older more obese patients with subjective daytime sleepiness. However, a direct association between concomitant sleep pathologies and NREM parasomnia‐related arousals was not confirmed in our study, with parasomnia behaviours being directly preceded by sleep‐disrupting pathology in only a minority of cases.

Up to 28% of patients with sleepwalking or sleep terrors have been reported to present with POD features (Schenck & Howell, 2013; Schenck et al., 1997). An updated classification included RBD‐SRED and RBD‐sexsomnia (AASM; Schenck & Howell, 2013). Subclinical RBD (RWA) can also coexist with NREM parasomnias (Schenck & Howell, 2013). RBD is considered a precursor of neurodegenerative diseases; RWA may also be relevant in this regard although more research is required (Boeve, 2010; Boeve et al., 2013; McCarter, St Louis, & Boeve, 2012). No data exist from observational studies covering the whole clinical spectrum of POD. In our study, 10.7% of the patients had features potentially suggestive of subclinical RBD with NREM parasomnia (incidental findings of RWA on vPSG); we did not classify these patients as having a POD diagnosis. Ultimately, 10% of our cohort of 512 consecutive patients were diagnosed with POD, highlighting a considerable overlap between these parasomnia conditions and pinpointing the importance of high clinical suspicion when assessing these patients in clinical settings. As outlined in the previous paragraph, sharing similarly increased percentages of concomitant sleep‐disrupting pathologies between confusional arousals and POD could explain at least partially the prevalence of POD, although further research is required to answer the question of whether there are any other pathophysiological relationships between NREM parasomnias and RBD.

The role of vPSG in NREM parasomnia does not seem to be confined to the simple identification of other sleep disorders. Within our cohort, vPSG had a relatively high diagnostic yield, providing data supporting the final diagnosis of parasomnia in 64.4% (330/512) of the patients. vPSG essentially confirmed the diagnosis in 31.4% (161/512) of the patients, capturing typical abnormal behaviours associated with spontaneous arousals from N3 sleep. Interestingly, irrespective of clinical phenotype, nearly all overt parasomnia events captured on vPSG in this cohort were confusional arousals. This may reflect a modifying effect of the sleep laboratory environment and equipment on parasomnia manifestations, highlighting the importance of clinical history in establishing the correct phenotypic diagnosis in patients with NREM parasomnia. Again, there is consistency between our findings and those from earlier, smaller cohorts. The currently published literature reports a diagnostic yield of vPSG in patients with parasomnias of approximately 60%–70% (Aldrich & Jahnke, 1991; Fois et al., 2015). In the previously mentioned study by Fois et al. (2015), vPSG provided decisive diagnostic information in 33.9% of patients and was supportive of the diagnosis in a further 26.6%. Nonetheless, the evidence base in this area otherwise remains relatively immature, largely consisting of small case series or case reports (Blatt et al., 1991; Guilleminault et al., 2005; Kavey et al., 1990).

Although the exact mechanisms behind NREM parasomnias are unknown, it is thought that priming factors that increase the amount of NREM3 sleep, and precipitating factors that increase the number of arousals from deep sleep are required to coexist in a genetically predisposed patient for a parasomnia event to occur (Schenck & Howell, 2013). Many of these factors were originally derived from case reports and clinical histories, but there is now a growing body of experimental evidence that supports their role in NREM parasomnia (Joncas, Zadra, Paquet, & Montplaisir, 2002; Pilon, Montplaisir, & Zadra, 2008; Zadra, Pilon, & Montplaisir, 2008). A possible priming factor was identified in more than one‐third of our patients, with stress and sleep deprivation accounting for the majority of these (81.4%). Although the identification of a potential priming factor may offer an attractive potential therapeutic avenue, further prospective interventional studies are needed to investigate the effect of modifying priming factors in NREM parasomnia patients.

### 4.1 Limitations

This is a retrospective study without a predetermined list of data collection for priming and precipitating factors, and limited data were available on family history and age of onset. Our data on concomitant physical sleep pathology apply to a sleep laboratory cohort and may not be applicable to the general population. We had relatively few cases of pure SRED and sexsomnia, so our data on these patients should be interpreted with caution. The lack of validated methods of diagnosing NREM parasomnia through vPSG questions the importance of abrupt arousals from NREM3 accompanied by more subtle behaviours, such as raising the head, sympathetic activation, such as tachycardia, or rhythmic delta activity on EEG, which are not specific to this disorder; consequently, it may be the case that the real diagnostic yield of performing vPSG in parasomnia patients may be significantly lower than our estimate. Although there are some data to support the notion that treating comorbid sleep pathology in parasomnia patients can improve control of parasomnia events (Drakatos et al., 2018; Guilleminault et al., 2005), it may be the case that the OSA and PLMS identified in this cohort are coincident phenomena rather than precipitating factors. Only a small minority of parasomnia events within our cohort were immediately preceded by either sleep‐disordered breathing or PLMS; a prospective study with more rigorous attention given to relationships between sleep‐disrupting pathology and parasomnia events could yield a stronger relationship. Finally, although we attempted to adjust our statistical analyses appropriately, we cannot discount the possibility that any apparently significant relationships we identified were type 1 errors contributed to by multiple comparisons.

## 5 CONCLUSION

vPSG has a high diagnostic yield in patients with NREM parasomnia and can aid diagnosis in unusual cases and cases complicated with RBD, and in differentiation from epilepsy. Clinicians should have a high clinical suspicion for physical sleep‐disrupting pathology in cases of NREM parasomnia, particularly older, male, obese and sleepy patients.

## CONFLICT OF INTEREST

None of the authors have any relevant conflicts of interest to declare.

## AUTHOR CONTRIBUTIONS

Conception and design: PD, RM, AJW, GDL, BDK. Data abstraction and analysis: PD, LM, SH, AN, EMD, RM, VG, LPC, ID, AB, SD, IR, DO'R, BDK. Review and approval of manuscript: all authors.
